# Treatment of Hemophilia

**Published:** 1896-09

**Authors:** 


### TREATMENT OF HEMOPHILIA.

Dr. A. E. Wright, Professor of Pathology in the Army Medical School, Netley, England, has demonstrated that chloride of calcium, in twenty to thirty grain doses, continued for some time, so increases the coagulability of the blood as to arrest hemophilic hemorrhage. He has been borne out in this observation by several other surgeons. The less soluble calcium salts act in a similar capacity when applied locally, and he has used with satisfactory results a paste of powdered chalk mixed with ½ per cent. solution of calcium chloride. Dr. Wright has himself confirmed Dr. Wickham Legg's statement that hemophilic children are frequently addicted to eating plaster, mortar, and such substances.

The inhalation of carbonic acid also has the power of increasing the blood coagulability, and as the calcium treatment may not always be successful, Dr. Wright advises the combination of the two. The following is a quotation from his paper in the *Lancet*, in which he also discusses the beneficial effects of cell nucleo-albumins in arresting hemorrhage:

Administrations of carbonic acid were resorted to whenever the epistaxis recurred. The administration of the gas was invariably followed by a prompt arrest of hemorrhage. The method of administration which was adopted consisted in leading a stream of carbonic acid into the nose through an india-rubber tube passed up well into the nostril. The carbonic acid was supplied from an ordinary Kipp's gasogene, such as can be purchased at any chemical apparatus dealer's for a few shillings. The patient was directed to hold his head forward in order that the blood should run out of the nostril along the india-rubber tube instead of trickling down the posterior nares. The coagulative effect of the carbonic acid was gauged by noting the rate at which the blood dripped from the tube. On several occasions this treatment was supplemented by a previous syringing out of the nostrils with ½ per cent. calcium chloride solution. In the case of epistaxis it is not necessary that the patient should inhale the gas; the local effect of the gas at the seat of hemorrhage will suffice. The same statement would hold good of a possible treatment of metrorrhagia by an administration of carbonic acid. It is, however, necessary to insist upon the fact that an excess of carbonic acid must be avoided if the method is to be effectual. My experiments upon animals have shown that the accelerating influence of carbonic acid gas upon blood coagulation is manifested only in presence of a sufficiency of oxygen. When this fact has been realized, it becomes evident that the inhalation of carbonic acid gas would be applicable to the treatment of hemoptysis. It is not necessary that the patient should be in any degree asphyxiated. Asphyxiation would militate against the efficacy of the method. The stream of gas should first be turned on very gently, so as to induce anesthesia of the mucous membranes. As soon as this has been effected very large quantities of the gas can be tolerated without discomfort. I have not had an opportunity of testing the method in a case of hemoptysis; it would appear, however, to be deserving of a trial, for it is a method which might result in an immediate arrest of hemorrhage. If the administration of carbonic acid were not effectual by itself, its coagulative effect might be enhanced by a previous administration of calcium chloride. It might, for instance, be applied an hour after the administration of thirty grains of calcium chloride by the mouth.

Finally, before dismissing the subject of the arrest of hemorrhage, it may be well to advert to the employment of solutions of cell nucleo-albumins as local applications to bleeding surfaces. The employment of physiological styptics of this kind would appear to be especially indicated in the treatment of hemophilia, for I have convinced myself by a somewhat extensive series of observations on the blood of hemophilic families, that the blood of hemophilic patients and of their female ascendants is characterized by a notable paucity of white blood-corpuscles, and especially by a relative paucity of the polynuclear white blood-corpuscles. In other words, hemophilic blood is deficient in the cellular elements which contribute the nucleo-albuminous element to the formation of fibrin. An addition of nucleo-albumins is, therefore, essential to the formation of a sound clot. The practitioner can always readily obtain a supply of these by mincing up a thymus gland, a testicle, or (if these cannot be obtained) a piece of gastric mucous membrane, in a little 1 in 500 solution of carbonate, and by filtering off the infusion, either immediately or after the lapse of a few minutes, through a piece of calico. I have employed such solutions of cell nucleo-albumins in two cases of hemophilic hemorrhage; in both cases the hemorrhage was taking place from cuts upon the hand. The result of the application of this physiological styptic was in each case the formation of an enormous mass of clot round the wound. In one of the cases bleeding continued for days under the clot (this was no doubt due to some dislodgment of the clot), and the skin became extensively macerated. I had in this case to clear away the clots and to arrest the hemorrhage by inhalations of carbonic acid combined with an application of lime salts. It is, perhaps, not too much to say that with these three methods of arresting hemorrhage at our disposal, very few, if any, cases of hemophilia ought to be allowed to succumb to their capillary hemorrhages.

